# Exploring Cereal Metagenomics: Unravelling Microbial Communities for Improved Food Security

**DOI:** 10.3390/microorganisms12030510

**Published:** 2024-03-02

**Authors:** Kedibone Masenya, Madira Coutlyne Manganyi, Tshegofatso Bridget Dikobe

**Affiliations:** 1 National Zoological Gardens, South African National Biodiversity Institute, 32 Boom St., Pretoria 0001, South Africa; 2 Department of Biological and Environmental Sciences, Sefako Makgatho Health Sciences University, P.O. Box 139, Pretoria 0204, South Africa; madiramanganyi@gmail.com; 3 Department of Botany, School of Biological Sciences, North-West University, Private Bag X2046, Mmabatho 2735, South Africa; tshegofatso.dikobe@nwu.ac.za

**Keywords:** metagenomics, food security, microbial communities, cereal crops

## Abstract

Food security is an urgent global challenge, with cereals playing a crucial role in meeting the nutritional requirements of populations worldwide. In recent years, the field of metagenomics has emerged as a powerful tool for studying the microbial communities associated with cereal crops and their impact on plant health and growth. This chapter aims to provide a comprehensive overview of cereal metagenomics and its role in enhancing food security through the exploration of beneficial and pathogenic microbial interactions. Furthermore, we will examine how the integration of metagenomics with other tools can effectively address the adverse effects on food security. For this purpose, we discuss the integration of metagenomic data and machine learning in providing novel insights into the dynamic interactions shaping plant-microbe relationships. We also shed light on the potential applications of leveraging microbial diversity and epigenetic modifications in improving crop resilience and yield sustainability. Ultimately, cereal metagenomics has revolutionized the field of food security by harnessing the potential of beneficial interactions between cereals and their microbiota, paving the way for sustainable agricultural practices.

## 1. Introduction

Cereal crops, such as wheat, sorghum, rice, maize, and barley, are staple food sources for a large portion of the global population [1,2]. These crops have emerged as sources for food security as well as biofuel production [1,3]. The cereal crops, like any other plant, host a diverse range of microorganisms, including bacteria, fungi, archaea, and viruses, collectively known as the plant microbiome [4]. These microorganisms form intricate communities within the soil, on plant surfaces, and even inside plant tissues. The cereals-microbe interactions are considered to be dynamic and can have beneficial, neutral, or detrimental effects on plant health [5]. They are ubiquitous in agricultural systems and play a diverse role in nutrient cycling, disease suppression, nutrient acquisition, defense against pathogens, and tolerance to abiotic stresses [6].

Interactions between the host plant and pathogens that are not beneficial have been classified as either predation or parasitism [7]. Parasitic/pathogenic microbes occur when microorganisms use plant resources such as water and nutrients to the detriment of the plant’s health, growth, and development [8]. Depletion of the plant’s resources reduces its fitness and increases its susceptibility to diseases, potentially leading to the host’s death [9]. Cereal production yield and quality are also constrained by many environmental factors, including certain diseases. Plant pathogens represent a constant and major food production constraint, with global crop losses estimated to be 20–30%, principally in food-deficit areas [10].

Harnessing the potential of these microbial communities is a promising approach to enhance crop production, reduce reliance on chemical inputs, and improve sustainability in agriculture [11]. Food security is a critical global challenge, especially in the face of population growth and climate change [1]. Ensuring a stable and sustainable food supply is critical for the well-being and survival of individuals and communities worldwide [11]. Agriculture plays a critical role in meeting this challenge, and understanding the complex interactions between plants, microbes, and their environments is crucial for enhancing food security [12].

Metagenomics, a powerful tool in microbial ecology and genomics, enables researchers to study microbial communities without the need for isolation and cultivation [13]. It entails direct sequencing of DNA extracted from environmental samples, providing a comprehensive view of the genetic diversity and functional potential of complex microbial communities [14]. Metagenomics has revolutionized our understanding of the complex relationships between microorganisms and their environments, including their interactions with plants in agricultural systems [15]. This technology has emerged in recent years as a valuable tool for deciphering the complex interactions between cereals and microbial communities [13]. Researchers can identify key microbial players and pathways involved in promoting plant health and productivity by investigating the genetic composition and functional potential of these microbial communities [16]. 

Furthermore, the integration of metagenomics, machine learning, and epigenetics represents a cutting-edge approach that holds great promise for unraveling the complex interactions within microbial communities and their host organisms [17,18]. Metagenomics provides a comprehensive view of the genetic composition of these communities, while machine learning algorithms offer powerful tools to analyze and extract meaningful patterns from vast amounts of data. By incorporating epigenetic information, such as DNA methylation patterns and histone modifications, researchers can gain a deeper understanding of how environmental factors influence gene expression and phenotype [19]. This integrated approach enables the identification of key microbial taxa, functional pathways, and epigenetic markers that play crucial roles in shaping host-microbe interactions, ultimately leading to novel insights and potential applications in agriculture. This knowledge can then be used to develop targeted crop improvement strategies, disease management strategies, and sustainable agricultural practices [4].

## 2. Microbial Communities Interaction with Cereal Plants

Microbiome-related metagenomics techniques have analyzed microbial communities; however, most of the studies conducted to date have largely dealt exclusively with bacterial communities [20,21]. It is also worth noting that in recent years, researchers have also largely focused on the belowground microbiome of all cereal crops, and not much has been performed on the aboveground microbiome [22]. The bacterial microbiome of cereal crops is largely dominated by *Proteobacteria*, *Actinobacteria*, *Firmicutes*, *Bacteriodetes*, *Acidobacteria*, and *Chloroflexi* [20]. The most commonly detected bacterial genera in the wheat, sorghum, and maize microbiome are *Pantoea*, *Pseudomonas*, *Rhizobium*, *Bacillus*, *Sphingomonas*, and *Stenotrophomonas* [23,24,25]. *Janthinobacterium*, *Pedobacter*, and *Erwinia* are bacterial genera that primarily dominate the wheat and barley phyllosphere [26,27]. Bacteria from the families *Comamonadaceae*, *Flavobacteriaceae*, and *Rhizobiaceae* dominated the barley root-enriched microbiota [28]. 

The studies targeting fungal communities have shown that cereals are dominated by Ascomycota and, to a lesser extent, by Basidiomycota [29,30]. The genera *Fusarium*, *Phoma*, *Pyrenophora*, *Alternaria*, and *Leptosphaeria*, which include well-known plant pathogens, are reported to dominate the epiphytic fungal communities of cereal seeds [31,32,33]. Pathogenic species commonly found in cereals include *Aspergillus* spp., *Botrytis cinerea*, *Colletotrichum* spp., *Epicoccum nigrum*, *Parastagonospora nodorum*, *Penicillium* spp., *Pyrenophora triticirepentis*, *Ramularia collocygni*, *Stagonospora* spp., and *Ustilago maydis* [34]. These species are known to be causal agents of major diseases in cereals and other plants [35,36].

The most economically important viruses identified using the metagenomics tools in wheat and barley are wheat streak mosaic virus (WSMV), triticum mosaic virus (TriMV), high plains wheat mosaic emaravirus (HPWMoV), soilborne wheat mosaic virus (SBWMV), barley yellow dwarf virus (BYDV), and cereal yellow dwarf virus (CYDV) [37,38]. The metagenomics tool also showed that maize was dominated by viruses from the *Betaflexiviridae* or *Tombusviridae* families. In addition, a novel DNA virus belonging to the *Geminiviridae* family was discovered in North American maize using the metagenomic approach [39,40]. Although the metagenomics tool has been used to characterize microbes, more research on fungi, viruses, archaea, and protists in cereal crops will be crucial to fully understand the relationship between plants, microbes, and the environment. The vital role of microbial communities in agriculture, including their impact on plant health, growth promotion, nutrient cycling, and soil health, and their detrimental effects have been extensively explored. The intricate role of microbes on crop plants is discussed further below. 

### 2.1. Beneficial Interactions

As agricultural production intensified over the last few decades, producers became increasingly reliant on agrochemicals as a relatively reliable method of crop protection, aiding in the economic stability of their operations [41]. However, increased use of chemical inputs leads to pathogen resistance to the applied agents as well as non-target environmental effects [42]. Furthermore, the rising cost of pesticides, particularly in less-affluent parts of the world, and consumer demand for pesticide-free food have prompted a search for alternatives. There are also a number of fastidious diseases for which there are few ineffective or non-existent chemical solutions [43]. As a result, biological control is being considered as an alternative or supplement to reducing the use of chemicals in agriculture [44,45]. 

A substantial proportion of plant-associated microorganisms is known for their antagonistic activity toward other microbes, including pathogens, due to their ability to produce hormones (Berg, 2009 [46]). The continued development of biological control agents (BCAs), which are used in agriculture to suppress pathogens, benefits greatly from the functional group of antagonists that are being studied. Some of the restrictions on biocontrol activity in the field can be lessened by using a combination of compatible biocontrol agents with different mechanisms of action. This combination can be effective in a wider range of climate conditions. Combinations like these may have synergistic effects that lead to increased protection, and a wider spectrum of diseases can be controlled [47,48]. This is accomplished through a variety of mechanisms, the most important of which are phosphorus-solubilizing bacteria, *Bacillus*, *Pseudomonas*, *Enterobacter*, and the fungi *Talaromyces aurantiacus* and *Aspergillus neoniger*, which are extremely effective at increasing plant available phosphorus in the soil, as well as improving crop growth and yield [49,50,51]. 

Under aerobic conditions, most iron is available in the soil in the insoluble form and is not readily available to plants, despite the fact that it is required for major physiological processes in plants, such as nitrogen fixation, photosynthesis, and respiration [52]. Microbes have evolved special mechanisms to chelate insoluble iron via the release of siderophores and the uptake of iron siderophore complexes via specific outer membrane receptor proteins [53]. The involvement of siderophores as a cooperative trait in *Pseudomonas* spp. has been well-established [54,55]. Mycorrhizal (symbiotic fungi) production of siderophores has also been reported [56]. 

Plant growth is also influenced by hormones like gibberellins, indole acetic acid (IAA), ethylene, and cytokinins. These hormones can be produced by the plant or by microbes associated with it, such as *Burkholderia phytofirmans* and certain fungi [57,58]. Plant-associated microbes can also have an impact on hormonal balance [59]. Ethylene is an important hormone effector, promoting plant growth at low levels while causing senescence, chlorosis, and leaf abscission at high levels. By lowering endogenous (1-aminocyclopropane-1-carboxylate (ACC) levels, bacteria containing ACC deaminase can reduce ethylene levels, resulting in increased root growth [60,61]. Because ethylene is a stress hormone, ACC deaminase-producing bacteria have the potential to protect plants from biotic and abiotic stress [62,63].

### 2.2. Non-Beneficial Interactions

Non-beneficial interactions between cereal plants and pathogens can be classified as either predation or parasitism [7]. Microorganisms that are parasitic or pathogenic exploit the plant’s resources, like nutrients and water, at the expense of the plant’s development, growth, and well-being [8]. The plant’s overall fitness is compromised, and it becomes more prone to diseases as a result of this resource depletion, which may cause the host plant to die [9]. Cereal yield and quality are hampered by environmental factors such as diseases, and more than 100 pathogens, including fungal, bacterial, and viral pathogens, can infect different parts of the plant in these crops [64]. Fungal diseases are more common in cereals than viral and bacterial diseases. The growth and productivity of cereal crops are seriously threatened by these pathogens. They may result in a number of symptoms and damage, including rot, wilting, leaf spots, and stunted growth. Severe instances of these diseases may result in notable reductions in grain yield and quality. Managing and controlling these diseases is crucial for maintaining healthy cereal crops. 

This can entail a number of strategies, such as using resistant cultivars, carrying out appropriate crop rotation, maintaining good sanitation, and using the right fungicides or other control measures when necessary. Researchers and farmers can create efficient management plans to reduce adverse effects and optimize the quantity and quality of cereal crops by having a thorough understanding of the variety and influence of these pathogens on cereals. Early detection and proper management strategies are crucial in minimizing the impact of these cereal pathogens on crop yield and quality. Table 1 below indicates some of the major cereal diseases; the symptoms can vary depending on the cereal crop, pathogen strain, and environmental conditions. 

## 3. Metagenomics: An Overview

Microorganisms represent two of the three domains of life, and about 99% of the microorganisms cannot be cultured by standard techniques [77]. Therefore, culture-independent methods are essential to understanding the genetic diversity, population structure, and ecological roles of the majority of microorganisms [78]. In this context, the advent of high-throughput next-generation sequencing (NGS) has revolutionized the field of microbial ecology and brought classical environmental studies to another level [79]. In fact, this type of technology has led to the establishment of the field of “metagenomics”, first coined in 1998 and defined as the direct genetic analysis of genomes contained within an environmental sample without the prior need for cultivating clonal cultures [15,77]. Next Generation Sequencing (NGS) has revolutionized the study of bacterial, viral, and fungal communities in plants using the metagenomics approach. 

### 3.1. Metagenomics Approaches for Studying Agricultural Microbiomes

A key component of metagenomics techniques for researching agricultural microbiomes is the examination of genetic material that has been directly extracted from environmental samples, such as soil and plant roots [80]. This approach can shed light on the composition, diversity, and functional potential of microbial communities associated with agricultural systems [14]. Targeted and shotgun metagenomics are two common techniques used in agricultural microbiome research [81]. The targeted/amplicon gene sequencing approach targets a specific region of the gene [82]. DNA metabarcoding uses a distinct pattern to identify living things; it is a short, highly variable, and standardized DNA region of about 700 nucleotides in length [83]. Metabarcoding has been widely used to gain a better understanding of evolutionary history and ecological biodiversity.

Metagenomics provides information about the taxonomic composition of microbial communities, making it possible to identify different microbial taxa [84]. Using gene-targeted sequencing to profile microbial communities is an easy and affordable way to profile the taxonomic makeup of microbes. However, because of the target gene’s conservation and the amplicon product’s length, its taxonomic resolution is restricted. Furthermore, targeted sequencing does not give us the microbe’s functional capacity [85,86]. 

The shotgun metagenomics technique entails sequencing every genetic molecule found in an environmental sample without first amplifying or focusing on any particular genes [87]. It enables the identification of microbial taxa that are both known and unknown [88]. Shotgun sequencing in microbiome studies can simultaneously identify and profile bacteria, fungi, viruses, and other types of microorganisms [81].

However, greater sequencing depth is required, which comes at a higher cost [89]. Despite the high costs, it can significantly improve taxonomic resolution and provide species-level assignment, whereas amplicon sequencing is limited to identifying genera [82]. Shotgun metagenomics also has the advantage of providing direct evidence of gene functional variation in the organisms present [90].

### 3.2. Utilization of Metagenome Studies to Identify Candidate Microbial Taxa and Genes

Metagenomics has enabled the identification of specific microbial species or groups that potentially benefit cereals (refer to Table 2). These studies provide valuable information on functional gene annotation within the cereal microbiome [91]. By comparing metagenomic sequences to reference databases, researchers can identify genes involved in various functions, such as nutrient cycling, plant growth promotion, disease suppression, and stress tolerance [92]. These functional genes shed light on the potential mechanisms underlying positive plant-microbe interactions [91]. Comparative metagenomic analysis involves comparing datasets from different cereal varieties or growth conditions [93]. By comparing the microbial composition and functional gene profiles between these datasets, researchers can identify specific microbial taxa and genes consistently associated with positive plant-microbe interactions. This approach also helps uncover candidate microbial taxa and genes likely to play crucial roles in cereal health and growth.

**Figure 1:**
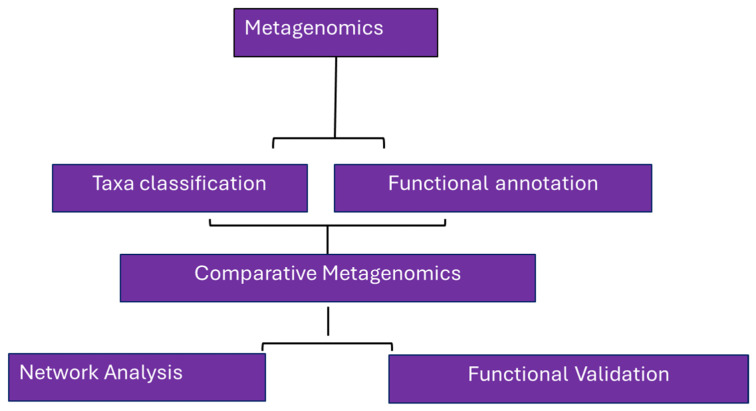
Metagenomics workflow to identify candidate taxa and genes.

To further explore the cereal microbiome, metagenome studies can be combined with network analysis. This approach allows researchers to identify highly connected microbial taxa and cereal genes within the cereal microbiome network [94]. By analyzing co-occurrence patterns and interactions between microbial taxa and cereal genes, key microbial species and potential candidate genes involved in positive plant-microbe interactions can be identified. This network-based approach helps prioritize microbial taxa and genes for further investigation and functional validation. Functional validation is crucial to confirm the roles of potential candidate cereal genes in positive plant-microbe interactions. This involves experimental techniques such as gene knockout or overexpression in cereals to assess their impact on microbial recruitment and plant health. Functional validation provides additional evidence for the involvement of candidate cereal genes in regulating the cereal microbiome [95]. By utilizing metagenome studies, researchers can uncover microbial taxa and candidate cereal genes associated with positive plant-microbe interactions. This knowledge can be further explored for the development of targeted interventions, such as microbial inoculants or genetic engineering approaches, to enhance cereal health, productivity, and sustainability. (See Figure 1 for the general workflow in identifying candidate taxa and genes).

**Table 2:** Metagenomics studies in identifying candidate taxa and genes.

Taxa Classification	Gene Identification	Host	Reference
Ascomycota, Basidiomycota, Mortierellomycota, Actinobacteria, Alphaproteobacteria, Bacteriodota, Gammaproteobacteria	Plant pathogen interactions. 3-Indol Acetic Acid (IAA) pathways, tryptophan metabolism, aminobenzoyl-glutamate. ACC deaminase pathway.	Wheat rhizosphere	[96]
Actinobacteria, Chloroflexi, Cyanobacteria, Firmicutes, Bacteroidetes, Proteobacteria, Acidobacteria, Gemmatimonadetes, Nitrospirae, Planctomycetes, Tenericutes, TM7	Iron metabolism. Ferritin1, Oxoglutarate/iron-dependent oxygenase Stabilizer of iron transporter SufD/Polynucleotidyl transferase.	Maize rhizosphere	[97]
Plant growth promoting taxa. Planctomycetes, Bacteroidetes, Verrucomicrobia, Cyanobacteria, Gemmatimonadetes, Chloroflexi, and Firmicute	Genes mitigating salt stress. Sulfur and glutathione metabolism bacterial chemotaxis, Sulfate reduction (*cysNC*, *cysQ*, *sat*, and *sir*), sulfur reduction (*fsr*), SOX systems (*soxB*), sulfur oxidation (*sqr*), organic sulfur transformation (*tpa*, *mdh*, *gdh*, and *betC*).	Grapevine rhizosphere	[98]
*Streptomyces renae*, *Streptomyces flavovariabilis*, *Streptomyces variegatus*, *Streptomyces chartreusis* and *Streptomyces cellvibrio*	Genes for metabolism of plant polysaccharides, iron, sulfur, trehalose, and vitamins, β-glucosidase Cellulose-hydrolyzing enzyme.	Tomato rhizosphere	[99]
Actinomycetia, Anaerolineae, Chloroflexia, and Nitrospira	Catalyzation of the transfer of oligosaccharides, dentification, nitrification, nitrate reduction genes, *ureB*, *ureA*, *glnA*, *nxrB*, *amoA_A*, *amoC_A*, *amoB_B*, *norC*, nirS, *nirK*, *nirD*, *narJ*, *narH*, *napC* *nirA*, *narC* nitrate reductase *(Anr)* and the gene *pmoA*.	Forest deep soil	[100]
Actinobacteria, Bacteroidetes, Firmicutes, and Proteobacteria	Carbohydrate metabolic processing, cell adhesion, pathogenesis, response to abiotic stimulus, and responses to chemicals.	Barley Rhizosphere	[101]
*Pseudomonas*, *Agrobacterium*, *Cupriavidus*, *Bradyrhizobium*, *Rhizobium*, *Mesorhizobium*, *Burkholderia*, *Cellvibrio*, *Sphingomonas*, *Variovorax* and *Paraburkholderia*	Plant-microbe and microbe-microbe interactions, nutrition acquisition, and plant growth promotion genes, *pqqB*, *appA*, *phnCEF*, *nrtABC*, *phoRPA*, *senX3*, *regX3*, *pmoA/amoA*, *ics*, *irp9*, *nagG*, *nagH*, *udC*, *nirK*.	Citrus rhizosphere	[102]
*Rhizophagus*, *Burkholderia*, *Trichoderma*, *Fusarium*, *Ochrobactrum phage POA1180*, *Blastococcus*, *Microvirga*, *Nocardioides*, *Geodermatophilus*, *Belnapia*, *Solirubrobacter*, *Arthrobacter*, *Mycobacterium phage Edugator*, and *Mycobacterium phage Kratio*	Not identified.	*Cleome pallida* (Desert plant) rhizosphere	[103]
*Kaistobacter* and *Rubrobacter Bacillus Nocardioides*, *Cellulomonas*, *Skermanella*, *Methylobacterium*, *Modestobacter* and *Aeromicrobium*, *Rhizobiales*, *Kaistobacter*, *Rubrobacter* or *Bacillus*	Metabolism of carbohydrate (especially C degradation) and membrane transporters. Carbohydrate degradation metabolism, carbohydrate synthesis, and its related energy metabolism.	Chickpea, wheat	[104]

### 3.3. Applications of Metagenomics in Enhancing Food Security

The availability of next-generation sequencing platforms enables metagenomic studies of bacterial, viral, and fungal disease complexes [105]. One of the key benefits of cereal metagenomics is the identification of beneficial microorganisms that can enhance crop productivity and resilience. Beneficial microbes can promote nutrient availability, improve soil fertility, stimulate plant growth, and provide protection against pathogens [106]. Furthermore, cereal metagenomics can help to develop strategies to mitigate the adverse effects of climate change on food security. Climate change-induced abiotic stressors, such as drought, salinity, and extreme temperatures, pose significant challenges to cereal crop production. By studying the microbiome’s role in conferring stress tolerance, researchers can identify microbial taxa and functional genes associated with stress resilience. This knowledge can be harnessed to develop microbial-based strategies for enhancing stress tolerance in cereal crops, thereby ensuring food security in the face of changing climatic conditions.

A number of papers have been published that describe the use of next-generation sequencing analysis of fungi infecting crop plants [107,108,109,110]. Metagenomic analyses have revealed the presence of plant growth-promoting bacteria, mycorrhizal fungi, and other beneficial microorganisms in the rhizosphere and endosphere of cereal crops. The use and demand for biopesticides and biofertilizers in agriculture and advancement in sequencing and metagenomics analysis have led to the discovery of beneficial microbes [111]. Understanding the functional potential of these microbial communities can lead to the development of novel biofertilizers, probiotics, and biocontrol agents to enhance cereal crop production [112].

Using next-generation sequencing (NGS) metagenomics approaches, Masenya et al. [25] compared the microbial communities between the resistant and the susceptible sorghum recombinant lines to identify any differences induced by pathogen infection. The results of the study revealed that pathogen infection led to distinct microbial community composition in the sorghum RILs. The presence of the pathogen influenced the abundance and diversity of specific microbial taxa in the leaf tissues. This suggests that pathogen infection plays a significant role in shaping the sorghum-associated microbiome. The study provides valuable insights into the interactions between pathogen infection and the sorghum-associated microbiome. Understanding the changes in microbial community composition induced by pathogen infection can contribute to the development of strategies for managing plant diseases in sorghum and potentially other crops. 

Similarly, in a study by Bziuk et al. [113], metagenomic analysis was conducted to investigate the impact of powdery mildew infection on the barley leaf microbiome. The researchers found that the presence of the pathogen altered the composition of the leaf microbiome, indicating a potential role of the microbiota in the defense response against powdery mildew. Metagenomic studies of crop and crop-related species may also be useful for the identification and surveillance of known and novel viral pathogens of crops. Lappe et al. [39] discovered novel viruses through the use of metagenomics.

This approach enabled the identification of beneficial microorganisms that naturally exist in the soil and have the ability to promote plant health and combat diseases [114,115,116]. It has also led to the identification of not only plant growth promotion but also disease suppression and/or other fitness-enhancing traits [117]. Several fungal and bacterial taxa in wheat plots were identified, suggesting the potential role of beneficial microbes in suppressing diseases [118]. Similarly, Terrazas et al. [101] discovered that the barley microbiota supports the assembly of a phylogenetically diverse group of bacteria that may be required to sustain plant performance.

The majority of the beneficial microbes identified were assigned to the bacterial phyla Proteobacteria and Firmicutes and the fungal phyla Ascomycota, specifically the genus *Trichoderma* [119,120]. Members of the genera *Pseudomonas* and *Bacillus*/*Paenibacillus* have also been frequently identified as plant-beneficial bacteria. For instance, inoculation with *Pseudomonas stutzeri* increased plant development and had a positive impact on bacterial community composition, particularly among diazotrophs and ammonia-oxidizers [121]. Therefore, metagenomics can be leveraged to develop innovative strategies for disease management and prevention, as the beneficial microorganisms can be isolated and utilized as biocontrol agents or sources of resistance genes. Consequently, enabling the researchers to develop sustainable and effective strategies to protect and enhance crop productivity.

### 3.4. Implications of Metagenomic Studies on Positive Plant Microbiome Interactions

Metagenomic studies demonstrating the presence of heritable taxa within microbiomes and their influence on positive plant microbiome interactions throughout evolutionary timelines have several implications. They can shed light on the co-evolutionary dynamics between plants and their associated microbiota [122]. By studying the heritable taxa within microbiomes, researchers can uncover long-term interactions that have shaped the genetic and functional diversity of both plants and microbes. This understanding helps unravel the complex and dynamic nature of positive plant microbiome interactions over evolutionary timescales [123].

These studies can identify conserved beneficial microbes that have co-evolved with plants over time. The heritable taxa may play key roles in nutrient acquisition, stress tolerance, and disease resistance. By characterizing their genomes and understanding their functions, researchers can develop targeted strategies to harness their beneficial effects for crop improvement and sustainable agriculture [124]. Metagenomics studies highlighting the presence of heritable taxa within microbiomes emphasize the importance of conserving and restoring natural plant-microbe interactions. Understanding the evolutionary history of positive plant microbiome interactions can guide conservation efforts, ensuring the preservation of beneficial microbial communities and their functions in natural and agricultural ecosystems [125]. 

**Figure 2:**
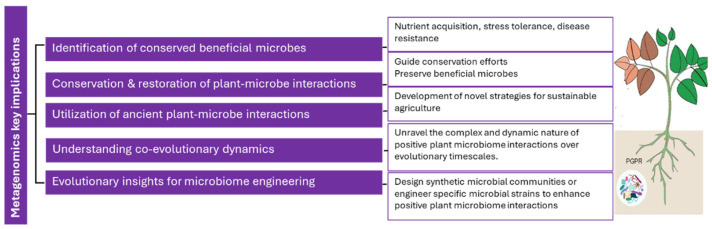
Positive plant microbiome interactions.

Metagenomics research can uncover ancient plant-microbe interactions that have persisted over evolutionary timescales. These interactions may involve heritable taxa that have co-evolved with specific plant lineages, providing unique benefits to their hosts. Understanding these ancient interactions can inspire the development of novel strategies for sustainable agriculture, including the utilization of ancestral microbial consortia or the reintroduction of specific microbial lineages to enhance plant health and productivity [122]. Metagenomic studies demonstrating the heritability of certain microbial taxa provide valuable insights for microbiome engineering efforts. By understanding the evolutionary history and genetic traits of beneficial microbes, researchers can design synthetic microbial communities or engineer specific microbial strains to enhance positive plant microbiome interactions. This knowledge can facilitate the development of targeted interventions for crop improvement and ecosystem restoration. In summary, metagenomic studies revealing the presence of heritable taxa within microbiomes and their influence on positive plant microbiome interactions throughout evolutionary timelines provide a deeper understanding of the long-term dynamics and potential applications of plant-microbe interactions. This knowledge can contribute to sustainable agriculture practices, conservation efforts, and the development of innovative strategies for crop improvement, refer to Figure 2.

## 4. Metagenomics and Integrated Epigenetics and Machine Learning Analysis

### 4.1. Practical Applications and Benefits of Employing Machine Learning in Epigenomic and Metagenomic Analysis

The intricate community of microorganisms associated with plant roots has been shown to be essential for plant health and overall fitness [126,127]. As a result, it has become a significant focus on plant-microbe interactions [114,115]. The plant’s response to these microbes is governed by an integrated network that includes not only the immune system but also other intrinsic biological systems within the plant itself [116,128]. Epigenetic factors, including DNA methylation, modifications to histone tails, chromatin accessibility, and DNA architecture, are closely linked to key cellular processes. When these components become dysregulated, it can lead to abnormal gene expression and disease [128]. The eukaryotic epigenome plays a crucial role in establishing and maintaining cellular identity and function.

DNA methylation is a well-known epigenetic modification that can be studied in metagenomics data. Several tools and pipelines have been developed to identify and quantify DNA methylation patterns in microbial genomes within metagenomics samples. The impact of defects in the RNA-directed DNA methylation (RdDM) pathway, which is responsible for establishing de novo DNA methylation, on plant resistance to *P. syringae* has been elusive [19,129]. Another study conducted in *Arabidopsis* demonstrated that Dicer-like (DCL) mediated siRNA production influences the assembly of the root microbiota, whereas downstream RdDM has no effect, suggesting that DCLs regulate the root microbiota through alternative epigenetic mechanisms [130]. As a result, it remains an important question whether and how epigenetic regulation can impact the assembly of root-associated microbial communities. It has been observed that simultaneous alterations in DNA methylation and histone modifications can be more effective, or even necessary, for epigenetic gene regulation [131,132]. An example of this is the *Arabidopsis* IBM1 (Increased in Bonsai Methylation 1) protein, which is involved in histone H3 lysine modifications.

In rice (*Oryza sativa*), histone methylation has been found to regulate the structure and composition of the root microbiota, particularly the hub species in the microbial network. Mutants DJ-jmj703 (JmjC domain-containing protein defective in histone H3K4 demethylation) and ZH11-sdg714 (defective in H3K9 methylation) exhibited significant differences in the root microbiota compared to their corresponding wild types at both the phylum and family levels. These differences included a consistent increase in the abundance of Betaproteobacteria and a decrease in Firmicutes [19]. These findings highlight the significant role of rice histone methylation in regulating the assembly of the root microbiota, shedding light on the connection between plant epigenetic regulation and root microbiota. In rice, DCL3 is responsible for processing 24-nt siRNAs (Small interfering RNA), which are involved in H3K9 methylation of histones [133].

Evidence is accumulating to suggest that epigenetic modifications are involved in the transcriptional regulation of plant disease resistance [17]. For example, DNA methylation in promoter regions can restrict the transcriptional expression of disease resistance genes, such as RMG1 and RLP43, in *Arabidopsis thaliana*. This limitation negatively impacts the plant’s resistance to bacterial pathogens like *Pseudomonas syringae* [134]. While previous research has examined the role of epigenetic regulation in plant disease resistance against specific pathogens, the potential impacts of epigenetic regulation on the composition of the root microbiota have remained uncertain. Notably, active DNA demethylation has been shown to play a positive role in plant resistance to pathogens such as *P. syringae* [135,136]. 

### 4.2. Machine Learning Coupled with Epigenomics in Identifying Differentially Methylated Regions

Recently, there has been development and reporting of machine learning techniques for the systematic detection of differentially methylated regions (DMRs) [137]. The potential to specifically modify the epigenome offers exciting possibilities for advancing our understanding of how epigenetic modifications function and for manipulating cell phenotypes in both research and therapeutic contexts. Epigenetic mechanisms play a crucial role in regulating gene expression in plants, responding to developmental processes and environmental cues, and ultimately impacting the plant’s overall characteristics [138]. Applied epigenetics is a rapidly evolving field of study, presenting new opportunities to enhance crop productivity. By combining epigenomics with machine learning, we can identify regions of the plant genome that undergo differential methylation during interactions with associated microbiota. However, the available data on the connection between epigenetics and plant-microbe interactions are currently limited [17,139].

Nevertheless, there are a few notable examples where a link has been established. One such example is the role of DNA methylation in the formation of root nodules during *Rhizobium* symbiosis in *Medicago truncatula* [139]. It has been found that a demethylase gene called DEMETER (AtDME) is involved in regulating a significant number of genes that are crucial for the differentiation of plant and bacterial cells, which is necessary for nodule organogenesis in symbiotic interactions [139]. An example of this is also demonstrated by Vigneaud et al. [140], who utilized epigenomics and transcriptomics approaches to investigate the interactions between poplar plants and the ectomycorrhizal fungus *Laccaria bicolor*. Their findings revealed that manipulating the expression levels of two demethylase genes (DML) and a chromatin remodeler (DDM1) influenced various parameters related to poplar root colonization by *L. bicolor*. Notably, they observed differential methylation in 288 transposable elements and 86 genes between hypomethylated mutant lines and wild-type poplar plants. This study serves as a proof of principle, shedding light on the role of the host plant’s epigenetic machinery during interactions with ectomycorrhizal fungi. It also raises intriguing questions about the potential influence of DNA methylation on plant interactions with endophytic fungi and bacteria. Epigenetic modifications have been observed in the seeds of *Geranium sylvaticum*, as well as the roots and leaves of *Geranium robertianum*, during arbuscular mycorrhizal (AM) symbiosis with the fungus *Funneliformis mosseae* [141]. 

Despite these advancements, there is still a need to further investigate the molecular mechanisms that govern plant-microbiome associations at a community level. It is paramount to identify the genes that enable plants to regulate the establishment of a beneficial root microbiota, as this knowledge will inform future breeding programs aimed at sustainably enhancing crop yield and quality. Deep learning and machine learning approaches hold immense potential for disease management, particularly in surveillance activities. These techniques are anticipated to facilitate precise monitoring of the host’s response and changes in microbiome composition, such as in the field of microbiome engineering [142]. By successfully integrating AI pipelines and multi-omics approaches, we can promise accurate isolation and identification of diverse microbes from various samples.

## 5. Metagenomics Workflow for Studying Agricultural Microbiomes

The metagenomics workflow typically consists of several steps, from sample collection to data analysis. Below is a high-level overview of the metagenomics workflow.

### 5.1. Sample Collection and DNA Analysis

The collection of environmental samples, such as soil, plant rhizosphere, and leaves from the agricultural system of interest, is the initial step. To preserve the microbial community, proper sampling techniques and storage conditions should be used [143]. Following sample collection, appropriate methods are used for DNA extraction. This step involves breaking open microbial cells and isolating the DNA for downstream analysis. 

### 5.2. Library Preparation and Sequencing

Prepare a sequencing library by fragmenting the extracted DNA and attaching sequencing adapters. Depending on the sequencing platform and study objectives, this step may involve additional steps like size selection or PCR amplification [144]. Perform high-throughput sequencing of the prepared library using next-generation sequencing platforms. This generates millions of short DNA sequence reads that represent the genetic material present in the sample [145].

### 5.3. Bioinformatics Analysis

**Figure 3:**
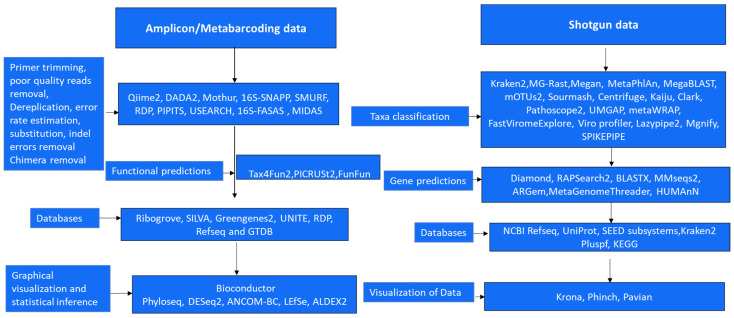
Workflow for high throughput metagenomics (amplicon and shotgun) data analysis [85,86,147,148,149,150,151,152,153,154,155,156,157,158,159,160,161,162,163,164,165,166,167,168,169,170,171,172,173,174,175,176,177,178,179,180,181,182,183,184,185,186,187,188,189,190].

The initial step is quality control checks in which adapter sequences, low-quality reads, and other artifacts are removed from the raw sequence data. Trimming, filtering, and merging paired-end reads may be part of this process [146]. The pre-processed sequences are examined to ascertain the microbial community’s functional potential and taxonomic composition. This can be accomplished using various bioinformatics tools and databases. Bioinformatics pipelines are available that can be used to predict functional annotations based on obtained sequences for functional analysis [85,147]. R and R studio are then used to interpret and visualize the results of taxonomic and functional analyses to gain insights into the composition and potential activities of the microbial community [148]. This could include generating taxonomic abundance profiles, diversity indices, functional pathway analysis, or other visualizations. Statistical analysis to identify significant differences or correlations in the microbial community composition or functional potential between different samples or treatments is conducted using R and R Studio [148]. See Figure 3 for a detailed overview of the bioinformatics pipelines for metagenomics studies. Some of the listed pipelines have been updated, including (version 4.0), Kraken (upgraded to Kraken 2), DIAMOND (version 2.0), Qiime2, MEGAN (version 6), HUMAnN (version 3), MetaGenomeThreader (version 1.6.2), and MetaPhyler (version 1.25) [147,149,150,151,152,153].

## 6. Challenges and Limitations in Metagenomics Studies

Metagenomics is a powerful approach to studying microbial communities and their genetic potential. Both metabarcoding and shotgun metagenomics are powerful techniques that allow for the study of microbial communities and have revolutionized the field of microbial ecology. However, there are certain limitations that researchers should be aware of, and therefore, we will discuss some of the key limitations of metabarcoding and shotgun approaches.

### 6.1. Sample Preparation Biases

Sample collection, DNA extraction, and library preparation processes can all introduce biases that affect the representation and diversity of microbial communities. The recovery and detection of specific microbial taxa can be influenced by factors such as sampling location, preservation methods, and DNA extraction protocols, potentially leading to skewed results. As a result, it is critical to ensure that standard procedures are followed.

### 6.2. Bias in DNA Extraction

The extraction of DNA from environmental samples can introduce biases. Different extraction methods may favor the recovery of specific microbial groups over others, resulting in an under- or over-representation of specific taxa in the metagenomic dataset. This bias may have an impact on the accuracy and completeness of the microbial community profile [191].

### 6.3. PCR Biases

The PCR amplification process used in metabarcoding can introduce biases and artifacts [192]. Certain taxa or DNA templates may be favored over others during PCR amplification, resulting in an overrepresentation or underrepresentation of certain microbial groups in the final sequencing data. This bias can affect the accuracy and representativeness of the microbial community composition [193].

### 6.4. Reference Database Limitations

Metabarcoding uses reference databases to assign taxonomic identities to the sequences obtained. These reference databases, however, may be incomplete or biased toward well-studied organisms [194]. This can lead to misidentification or underrepresentation of certain taxa, particularly for less well-known or novel species. Furthermore, the composition and quality of reference databases can differ across ecosystems or regions, affecting the accuracy of taxonomic assignments [195].

### 6.5. Detection Limits

Metabarcoding may be limited in detecting rare or low-abundance taxa within a microbial community. This is especially important when studying complex ecosystems with high microbial diversity or when analyzing low biomass samples. Due to sequencing depth limitations and PCR amplification biases, rare taxa may be missed or underestimated [196].

### 6.6. Taxonomic Resolution

The limited taxonomic resolution provided by metagenomics metabarcoding is one of its main limitations. Metabarcoding typically involves amplifying and sequencing a specific genomic region. These regions, however, may not have enough resolution to accurately classify and identify species [197]. Unless the long fragment approach is used, which produces Amplicon Sequence Variants (ASVs), this can result in the grouping of closely related species into the same operational taxonomic unit (OTU), making it difficult to distinguish their individual ecological roles [198].

### 6.7. Fragmented Genomes

The sequencing and assembly of DNA fragments from environmental samples is the foundation of shotgun metagenomics. This can result in fragmented genomes, making accurate reconstruction of complete genomes for individual microorganisms difficult [199]. This limitation can make it difficult to analyze microbial functional potential and identify specific genes or pathways.

### 6.8. Difficulty in Functional Annotation

While metagenomics provides information about the genetic potential of microbial communities, functional annotation of the sequences obtained can be difficult. Assigning specific functions to genes or predicting metabolic pathways from metagenomic data is a difficult task that frequently necessitates further experimental validation or integration with other omics approaches [200].

### 6.9. Computational and Storage Requirements

Metagenomic data analysis necessitates substantial computational and storage resources. The large volume of sequencing data generated in metagenomics studies can present difficulties in data management, computational infrastructure, and analysis pipelines [79,201]. Adequate computational resources and expertise are required to effectively handle and process metagenomic datasets.

Despite these limitations, metagenomics is still a useful tool for investigating microbial diversity, community structure, and functional potential. We now have a much better understanding of microbial communities thanks to this technique. Researchers can maximize the utility of metagenomics and gain valuable insights into microbial ecosystems by understanding these limitations and employing appropriate controls and validation strategies. However, it is critical to understand the limitations so that the constraints can be carefully considered and addressed.

### 6.10. Challenges Associated with Identifying Primary Cereals Loci

It has been discovered that host genetics play a significant role in determining the composition of the plant microbiome. However, it remains challenging to identify the specific genetic loci that control microbial selection [202]. While there is consistent evidence of the interaction between host genetics and plant microbiome composition, pinpointing the genetic elements responsible for host-genotype-dependent microbiome acquisition and assembly in plants is still a difficult task. Some studies have started to explore the impact of individual host genes on microbiome composition based on prior hypotheses of gene involvement [203,204]. However, these studies are limited to a small subset of plant genes that are predicted to be involved in microbiome-related processes. 

Additionally, many plant traits that are expected to influence microbiome composition and activity, such as root exudation and root system architecture [205], are complex and potentially regulated by a large number of genes. Therefore, there is a need for alternative large-scale and unbiased methods to identify the genes that control the host-mediated selection of the microbiome. Genome-wide association studies (GWAS) offer a powerful approach to map the loci associated with complex traits in genetically diverse populations. GWAS can be a valuable tool in identifying microbes that are sensitive to host genotypes and linking them to the genetic loci that influence their colonization. Microbiome genome-wide association studies (mGWAS) have been used to understand the interaction between host genetic variation and the microbiome in *Arabidopsis thaliana* [6,206].

**Figure 4:**
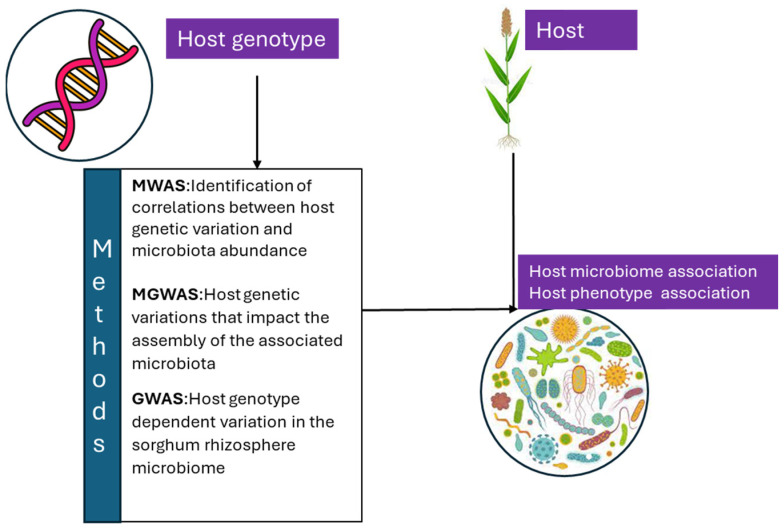
Approaches to study host genetics and plant microbiota.

However, understanding the factors that shape host-microbe interactions and their impact on phenotypes is still limited. Additionally, the beneficial effects of bacterial strains on hosts are often specific to certain cultivars and species, making it challenging to apply them universally. Therefore, it is crucial to uncover the genetic variability for agronomic traits, which can expand the gene pool for breeding programs and improve the effectiveness of genetic engineering for stress tolerance. While some loci associated with specific traits have been identified through GWAS in millet, the loci related to plant growth or yield remain unknown. In a previous study, the microbial composition of the root zone microbiota in millet and its correlation with yield traits through extensive sampling and analysis was examined [202]. The genetic variations associated with agronomic traits in foxtail millet were identified. Although GWAS has been widely used to understand plant phenotypes, its ability to capture complex agronomic traits is limited. To address this challenge, a new approach called microbiome-wide association studies (MWAS) has emerged, which has successfully identified gut microbial markers for complex traits in human cohorts. However, MWAS in plants has been relatively scarce. Therefore, combining GWAS, MWAS, and mGWAS can provide valuable insights into precision agriculture, particularly regarding genotype-dependent microbial effects in cereals, see Figure 4.

## 7. Reliability and Reproducibility

Metagenomics is an effective tool for understanding and harnessing the potential of crop microbial communities. However, it is important to note that challenges still exist, such as potential biases introduced during DNA extraction, PCR amplification, and sequencing [207]. High-throughput sequencing data can also be affected by different technical errors. As a consequence, the reproducibility of experiments can be weakened and consequently results in falsely identified microorganisms [208]. Nevertheless, the reliability and reproducibility of metabarcoding and metagenomics in microbial profiling have been extensively studied and are generally considered to be high [209]. Technological advancements and standardization efforts are continuously improving the reliability and reproducibility of metabarcoding and metagenomics in microbial profiling.

Efforts have been made to ensure the reproducibility of these techniques and include standardized protocols, such as DNA extraction methods. Errors can be identified when comparing the results obtained using different methods of DNA extraction, and good reproducibility is achieved using one method [210]. The PCR amplification primers have been developed to minimize variation between studies. However, the more primers are used in parallel, the more PCR artifacts occur. Nevertheless, bioinformatics approaches are available to remove the artifacts introduced during library preparation. The sequencing results obtained on different platforms are characterized by low reproducibility with each other because of differences in systematic errors on the different platforms [211]. Increasing the sequencing depth, as measured by the number of reads per sample, is the primary method for improving reproducibility [90]. Preferably, all the sequences of the experiment should be obtained on the same platform (at least in the same study). 

A poor choice of the clustering algorithm amplicon sequencing variants or operational taxonomic units, as well as identity threshold, can be detrimental to the performance. It is fundamentally important that all comparisons must be made only between results obtained using the same clustering method [212]. Allali et al. [211] demonstrated that the analysis of the same sample with different mathematical packages and/or different sequencing platforms leads to data irreproducibility.

## 8. Contribution of Large-Scale Cereal Microbe Genetic Datasets to the Advancement of Knowledge

The contribution of large-scale cereal microbe genetic datasets to the advancement of knowledge is significant and far-reaching. These datasets provide valuable insights into the genetic makeup and interactions of microorganisms associated with cereal crops [22]. By analyzing the genetic profiles of these microbes, scientists can identify specific genes or pathways responsible for promoting plant growth, suppressing pathogens, or improving nutrient availability [202]. This knowledge can then be used to develop targeted strategies for enhancing crop productivity and reducing the reliance on chemical inputs. Furthermore, large-scale cereal microbe genetic datasets enable researchers to understand the complex interactions between microorganisms and their cereal hosts [213]. 

By studying the genetic information of both the microbes and the plants, scientists can unravel the intricate networks of molecular communication and signaling that occur between them [214]. This knowledge can help in the development of novel approaches for managing plant diseases, such as using beneficial microbes as biocontrol agents or engineering crops with enhanced disease resistance. In addition, these datasets contribute to our understanding of the evolution and diversity of cereal-associated microorganisms [215]. By comparing the genetic sequences of different microbial strains, researchers can trace their evolutionary history and identify patterns of genetic variation. This information can shed light on the origins of specific traits or adaptations and provide insights into the mechanisms driving microbial diversity. 

Overall, the contribution of large-scale cereal microbe genetic datasets to the advancement of knowledge is immense. These datasets provide a wealth of information that helps us understand the complex interactions between microorganisms and cereal crops, identify beneficial microbes, unravel molecular communication networks, study microbial evolution, and discover novel genes and pathways. By harnessing this knowledge, we can develop innovative strategies for improving crop productivity, sustainability, and resilience in the face of environmental challenges.

## 9. Advances Facilitated by HTS Technologies in Understanding Cereals-Associated Microorganisms

### 9.1. Long Read Sequencing

Generating metagenome-assembled genomes (MAGs) from a shotgun metagenome dataset is one of the best techniques for investigating these prokaryotic phantoms [216]. For these assembly-focused studies, PacBio long-read and Oxford Nanopore sequencing technologies offer superior performance, enabling researchers to generate more MAGs and more circular single-contig MAGs than short-read sequencing alternatives [217,218]. Moreover, long-read technologies are now capable of providing methylation patterns, which can help establish associations between multiple replicons, potentially revealing the presence of multiple chromosomes and plasmids within a genome [219]. Despite the advantages, barriers to long-read sequencing still exist, causing short-read platforms to continue dominating much of the metagenomics sequencing market. This has led to the rise of technologies such as synthetic long reads and Hi-C that use alternative library preparation methods and short read sequencers as alternatives to long-read sequencing.

### 9.2. Hi-C

An alternative approach to enhance genome assembly is through the use of Hi-C. This method capitalizes on the ability to link co-located DNA during library preparation. Initially employed to improve genome assembly for larger genomes, Hi-C has more recently been applied to metagenomics studies [220]. During library preparation of metagenomic samples, the DNA within bacterial cells is cross-linked by binding to surrounding proteins. Subsequently, restriction enzymes are used to cut the DNA, and ligation is performed. This process enables DNA fragments from the same cell to stick together [221]. After sequencing, the reads are computationally assigned to the respective cell, thereby improving the generation of MAGs and enabling the linkage of plasmid and phage DNA to specific host strains. Commercial kits and analysis pipelines are available, with Phase Genomics being a major contributor in this field. Hi-C is a powerful technology that provides direct and quantitative measurements of DNA sequences from shotgun sequencing [220]. It yields a higher number of high-quality genomes and captures insights at the strain level. This technique significantly enhances the quality and reliability of assembled genomes from shotgun metagenomic samples, facilitating accurate identification of plasmids, phages, antibiotic resistance genes, and other mobile genetic elements within host cells [222]. The improved assembly and strain-level genomic resolution of the microbiome will aid in tracking genes associated with antibiotic resistance and disease prevention. 

### 9.3. CRISPR

Recently, gene-editing approaches such as clustered regularly interspaced short palindromic repeats (CRISPR) have been utilized to enhance host systemic-induced resistance against phytopathogens [204]. Additionally, these techniques have been employed to expedite the domestication of wild crops, allowing for the reintroduction of beneficial plant growth-promoting (PGP) traits from the rhizosphere microbiome of wild relatives of specific crops [223,224,225]. These strategies have the potential to address challenges related to the persistence of microbial bio-inoculants and concerns regarding the containment of genetically modified rhizosphere microbiomes. By combining signal recognition with containment techniques, these approaches offer promising solutions.

### 9.4. Machine Learning

There are two main challenges in microbiome data analysis, which include species identification and model selection. Computational models that analyze microbiome data can aid in the association analysis between the microbiome and plant hosts. This is because microbiome data obtained through next-generation sequencing (NGS) is complex, sparse, noisy, and high-dimensional. To meet these technical demands, researchers have turned to machine learning-based methods, such as random forest (RF), to study the impact of the microbiome on plant growth [226]. This is due to the rapid development of machine learning (ML) techniques, which have resulted in significant advancements in microbiome research. These techniques have allowed researchers to delve into the data-rich world of microbiome analysis. 

## 10. Future Directions and Emerging Technologies

To provide a comprehensive analysis of crop microbe research, various omics tools and techniques are available for the analysis of agricultural microbiome research. The metatranscriptomics approach entails sequencing and analyzing RNA molecules found in environmental samples. It provides insights into the active microbial community and their gene expression patterns, assisting in the understanding of the agricultural microbiome’s functional activities and metabolic processes. Another approach is metaproteomics, which involves the identification and quantification of proteins expressed by the microbial community in a given sample. It provides information on microbial communities’ functional activities and interactions with the agricultural environment. We also have metabolomics, which is the study of small molecules produced by microbial communities known as metabolites. It details the metabolic activities and chemical interactions of microbes in the agricultural microbiome.

Although each approach provides valuable information separately, when combined, they paint a more comprehensive picture and can hold the key to an in-depth understanding of microbiomes [227]. These omics approaches can enhance metagenomics research by providing comprehensive data on functional potential, metabolism, expressed proteins, and microbial community activity. Scientists can identify potential targets for genetic engineering or the development of new crop protection products by examining the functional genes and pathways found in microbial communities. 

The integration of omics tools in microbial studies allows for a more comprehensive understanding of microbial communities, their functional potential, and their responses to environmental changes. It enables researchers to identify key players and processes within a community, uncover novel metabolic pathways, and discover potential biomarkers for disease diagnosis or environmental monitoring. Ultimately, the integration of omics tools enhances our understanding of microbial ecosystems and opens new avenues for biotechnological applications and interventions. As a result, combining these omics approaches can help researchers gain a better understanding of the functional capabilities, metabolic pathways, and interactions within microbial communities. This multi-omics approach allows for a more in-depth exploration of microorganisms’ complex dynamics and ecological roles in a variety of environments, including the phylosphere, soil ecosystems, and aquatic habitats. 

## 11. Conclusions

Cereal metagenomics offers a promising avenue for enhancing food security by leveraging the beneficial interactions between cereal crops and their associated microbiota. By unraveling the complexities of the cereal microbiome, researchers can identify novel microbial resources and functional genes that can be utilized to improve crop productivity, nutrient uptake, and stress tolerance. The integration of cereal metagenomics with conventional breeding and agronomic practices holds significant potential to address the challenges of global food security in a sustainable and environmentally friendly manner.

Understanding the interactions between cereals and microbial communities is of paramount importance for improving their productivity and resilience to environmental stresses. Microbes associated with cereal crops can contribute to nutrient acquisition, pathogen suppression, and tolerance to abiotic stresses, thereby enhancing crop growth and overall yield. Therefore, the metagenomics approach has direct implications for food security. Integration of omics tools for microbial studies involves combining multiple high-throughput technologies to gain a comprehensive understanding of microbial communities at various levels. By integrating genomics, transcriptomics, proteomics, and metabolomics, researchers can obtain a holistic view of the genetic potential, gene expression, protein profiles, and metabolic activities of microorganisms within a community. This will also facilitate our understanding of the interplay between microbiomes and cereal crops, as it is central to the elucidation of the response to biotic and abiotic stress in agriculturally important crops.

## Figures and Tables

**Table 1:** Fungal and bacterial pathogens of cereals.

Cereal Diseases	Bacteria/Fungi	Symptoms	Cereal Crops	References
Fusarium head blight	*Fusarium graminearum*	Bleached or discolored spikelets, premature ripening, and pink or orange fungal spore masses on infected heads.	Wheat, rice, barley	[65]
Bacterial leaf blight	*Xanthomonas campestris*	Symptoms include water-soaked lesions with yellow halos on leaves. Lesions may expand and coalesce, leading to leaf wilting and plant death.	Wheat, sorghum, barley crops	[66]
Common charcoal root rot	*Cochliobolus sativus*, *Macrophomina phaseolina*	Symptoms include dark brown to black lesions on the roots and lower stem. Infected plants may exhibit stunted growth, reduced tillering, and wilting.	Sorghum, barley, wheat	[67]
Tan spot	*Pyrenophora tritici-repentis*	Symptoms include tan or brown necrotic lesions with yellow halos on leaves. Lesions may coalesce, leading to extensive leaf damage and reduced grain yield.	Wheat, maize, sorghum	[68]
Fungal leaf blight	*Exserhilum turcicum*	Large cigar-shaped lesion oriented lengthwise along the leaf.	Sorghum, wheat, maize	[69]
Bacterial leaf spot	*Pseudomonas syringae*	Water-soaked spot lesions on leaves.	Sorghum, wheat	[70]
Bacterial leaf stripe	*Burkholderia andropogonis*, *Pseudomonas andropogonis*, *Pseudomonas sorghicola*	Characterized by long, narrow stripes that can vary from red to black.	Maize, wheat, oats, sorghum	[71]
Anthracnose	*Colletotrichum sublineolum*	Small, circular, elliptical, or elongated spots.	Sorghum, maize, Barley, rye, oats	[72]
Leaf Scald	*Rhynchosporium secalis*	Elongated, brown lesions with yellow halos on leaves. Severe infections can lead to premature leaf death and reduced grain yield.	Barley	[73]
Grain molds	*Fusarium* spp., *Curvularia lunata*, *Alternaria alternata*, *Phoma sorghina* and *other fungi*	Pink, orange, or white seeds found on the infected heads.	Sorghum, maize, Wheat, oats	[74]
Powdery mildew	*Blumeria graminis*	White or gray powdery fungal growth on leaves, stems, and panicles. Infected plants may exhibit stunted growth, reduced photosynthesis, and premature senescence.	Sorghum, maize, Barley, oats	[75]
Rust	*Puccinia purpurea*	Reddish-brown pustules on stems, leaves, and spikelets. Infected plants may exhibit stunted growth, chlorosis, and reduced grain yield.	Sorghum, maize, Barley, oats	[76]

## Data Availability

Not Applicable.
